# Low-Voltage Solution-Processed Zinc-Doped CuI Thin Film Transistors with NOR Logic and Artificial Synaptic Function

**DOI:** 10.3390/nano13162345

**Published:** 2023-08-15

**Authors:** Xiaomin Gan, Wei Dou, Wei Hou, Xing Yuan, Liuhui Lei, Yulan Zhou, Jia Yang, Diandian Chen, Weichang Zhou, Dongsheng Tang

**Affiliations:** Synergetic Innovation Center for Quantum Effects and Application, Key Laboratory of Low-Dimensional Quantum Structures and Quantum Control of Ministry of Education, Hunan Normal University, Changsha 410081, China; gxm012399@163.com (X.G.); whouhouwei@163.com (W.H.); yx222198@163.com (X.Y.); lhlei182@163.com (L.L.); yilanchow@hunnu.edu.cn (Y.Z.); m18890724966@163.com (J.Y.); chendiandian@hunnu.edu.cn (D.C.)

**Keywords:** thin film transistors, low voltage, electric-double-layer, NOR logic operation, Zn-doped CuI, artificial synaptic

## Abstract

Low-voltage Zn-doped CuI thin film transistors (TFTs) gated by chitosan dielectric were fabricated at a low temperature. The Zn-doped CuI TFT exhibited a more superior on/off current ratio than CuI TFT due to the substitution or supplementation of copper vacancies by Zn ions. The Zn-doped CuI films were characterized by scanning electron microscope, X-ray diffraction, and X-ray photoelectron spectroscopy. The Zn-doped CuI TFTs exhibited an on/off current ratio of 1.58 × 10^4^, a subthreshold swing of 70 mV/decade, and a field effect mobility of 0.40 cm^2^V^−1^s^−1^, demonstrating good operational stability. Due to the electric-double-layer (EDL) effect and high specific capacitance (17.3 μF/cm^2^) of chitosan gate dielectric, Zn-doped CuI TFT operates at a voltage below −2 V. The threshold voltage is −0.2 V. In particular, we have prepared Zn-doped CuI TFTs with two in-plane gates and NOR logic operation is implemented on such TFTs. In addition, using the ion relaxation effect and EDL effect of chitosan film, a simple pain neuron simulation is realized on such a p-type TFTs for the first time through the bottom gate to regulate the carrier transport of the channel. This p-type device has promising applications in low-cost electronic devices, complementary electronic circuit, and biosensors.

## 1. Introduction

As a key element of flat panel displays, TFTs have been extensively researched [1]. There are fewer investigations into the creation of p-type oxide TFTs compared to n-type oxide TFTs because the high-quality film growth of p-type oxide semiconductors is complicated [2]. Several studies have been recently published on the production of p-type oxide TFTs utilizing conventional vacuum coating techniques. Hye-Mi Kim et al. fabricated two-step crystallization SnO-based TFTs based on atomic layer deposition [3]. Soo Hyun Kim et al. investigated the effect of annealing temperature on the quality of SnO thin films and fabricated high performance SnO TFTs with an on/off current ratio of 2 × 10^6^ [4]. Cheng Wei Shih et al. fabricated a p-type SnO TFT with a subthreshold slope of 140 mV/dec through the introduction of oxygen during magnetron sputtering. [5] However, high equipment costs and relatively small deposition areas limit the potential use of traditional vacuum technology in large-area electronic equipment [6]. In 2013, Sang Yun Kim et al. first proposed a p-type Cu_2_O TFT with an on/off current ratio of ~1 × 10^2^ and a field effect mobility of 0.16 cm^2^V^−1^s^−1^ based on solution treatment [7]. Since then, several studies have been reported successively on solution-treated p-type oxide TFTs, such as Ni_x_O TFTs, Cu_x_O TFTs, and SnO_2_ TFTs [8,9,10]. In 2016, Jaewon Jang et al. fabricated a CuO TFT based on copper acetate (II) precursors annealed at lower than 500 °C [11]. However, because of the localized hole transport path in the valence band maximum (VBM) and the strong self-compensation during doping, p-type oxide semiconductors exhibit poor electrical properties [12,13]. Recent theoretical studies have shown that anions with larger p orbitals and less electronegativity are more favorable for achieving delocalized VBM than O^2-^, which leads to wide research on metal halides in the manufacture of p-type semiconductors [14]. CuI is considered the most potential candidate on account of its significant conductivity of 238 S·cm^−1^, wide bandgap (Eg) of ~3 eV, and high doping capacity [15,16]. In addition, the high optical transmittance and low preparation temperature (<100 °C) of CuI provide a new path for transparent electronic devices. In 2020, Ao Liu et al. first reported p-type Zn-doped CuI TFTs on SiO_2_/Si substrates and confirmed that Zn^2+^ was the optimal n-type dopant [17].

N-type semiconductor devices are widely studied for integrated circuits and flat panel displays because of their excellent electrical properties. In 2020, Wanpeng Zhao et al. fabricated a high-gain transparent inverter based on deuterated ZnO TFTs. The maximum voltage gain could reach −310 V/V, which could be used as a reliable candidate for a common source amplifier [18]. Sun Yang et al. fabricated an 8-inch flexible miniature LED display based on indium gallium zinc oxide (IGZO) TFTs with a top gate structure [19]. The application of p-type devices in numerous fields is extremely rare. In addition, artificial synaptic research based on n-type semiconductor devices has also received extensive attention in recent years. The human brain is a highly intelligent and complex information processing system that differs from the Von Neumann structure. The calculation and storage of data processed by the Von Neumann structure are distributed. However, the computation and storage of information processed by the human brain are parallel. The neuronal system of the human brain includes neurons and synapses predominantly. Neurons are connected to each other through synapses to constitute complex three-dimensional structures. In the nervous system, there is no clear boundary between memory and information calculation, and each synapse of every neuron stores and processes information synchronously. Therefore, synapses and neurons have been extensively studied in neuronal devices. Wan Qing et al. fabricated indium gallium zinc oxide (IGZO) nanofiber photosynaptic transistors and realized high-precision neural morphology calculations [20]. Dongliang Jiang et al. reported a solution-processed In_2_O_3_ TFT with memory and learning functions attained by light stimulation [21]. However, related research based on p-type devices is not common.

Moreover, resource consumption has become an issue that people must pay attention to, especially in the power supply system and large-scale equipment production. Compared with traditional vacuum coating technology, the preparation and application of large-area deposition electronic devices based on the solution method are particularly important. As a biopolymer, chitosan has the advantages of biodegradability and being naturally pollution-free, which can greatly reduce environmental pollution and waste of resources. In addition, chitosan is formed by N-deacetylation of chitin and is easily soluble in acidic aqueous solutions [22]. In the gated transistor prepared by using chitosan as the dielectric, the electric double layer is formed at the interface after the source electrode and gate electrode apply voltage, which leads to the accumulation of carriers on one side of the channel near the gate dielectric. More importantly, chitosan has a high dielectric constant, so the device can work at ultra-low voltage. It facilitates the reduction in energy consumption and the production of portable electronic equipment.

In this study, low-voltage Zn-doped CuI TFTs gated by chitosan dielectric have been fabricated on FTO glass substrates. A large specific gate capacitance of 17.3 μF/cm^2^ was obtained in the chitosan film owing to the EDL effect, which allows TFTs to work at low voltage. The TFTs showed good performance with a field effect mobility of 0.40 cm^2^V^−1^s^−1^, a subthreshold swing of 70 mV/decade, and an on/off current ratio of 1.58 × 10^4^. The threshold voltage is −0.2 V. In addition, NOR logic was realized on such p-type Zn-doped CuI TFTs first. Importantly, the basic operating principle of artificial synaptic simulation based on TFT was analyzed at length. Excitatory postsynaptic currents (EPSC) and inhibitory postsynaptic currents (IPSC) could be achieved on this Zn-doped CuI TFT. A simple human brain pain simulation based on the TFT was also obtained.

## 2. Materials and Methods

### 2.1. Synthesis of Precursor Solution

The 0.1 M CuI precursor solution was prepared by dissolving CuI in acetonitrile and stirring at room temperature for 30 min. The Zn-doped CuI precursor solution was prepared by mixing CuI and ZnI_2_ in acetonitrile and stirring for 30 min at room temperature. The molar ratio C = N_Zn_/(N_Zn_ + N_Cu_) was set at 1% by fixing N_Zn_ + N_Cu_ = 0.1 M. The chitosan powder was dissolved in deionized water and acetic acid solution to make the chitosan precursor solution (mass ratio M_deionized water_:M_chitosan powder_:M_acetic acid solution_ = 40:1:1). To form a stable solution, the chitosan precursor solution was stirred for 1 h and rigorously mixed using a sonicator for 40 min. Then, the precursor solutions were stored in a sealed room temperature environment. The Zn-doped CuI precursor solution was stirred for 10 min before the experiment.

### 2.2. Preparation for Characterization of Zn-Doped CuI Film

Sample 1 was prepared for X-ray diffraction and X-ray photoelectron spectroscopy characterization of the Zn-doped CuI film. Firstly, the SiO_2_/Si substrate was sequentially sonicated in deionized water and alcohol for 45 min. Secondly, we deposited the Zn-doped CuI precursor solution on the SiO_2_/Si substrates by spin coating at 3000 rpm for 20 s and then annealing at 80 °C for 20 min. Sample 2 was prepared for scanning electron microscope characterization of the Zn-doped CuI film. Firstly, the FTO glass substrate was sequentially sonicated in deionized water and alcohol for 45 min. Secondly, the chitosan precursor solution is deposited on the FTO glass substrates by spin coating at 3000 rpm for 20 s and then annealing at 80 °C for 30 min. Finally, we deposited the Zn-doped CuI precursor solution on the SiO_2_/Si substrates by spin coating at 3000 rpm for 20 s and then annealing at 80 °C for 20 min.

### 2.3. Fabrication of CuI TFT and Zn-Doped CuI TFTs

First, the Zn-doped CuI precursor solution was stirred at room temperature for 10 min to obtain a uniform and stable liquid before TFT fabrication. Second, the chitosan precursor solution was deposited on the FTO glass substrates by spin coating at 3000 rpm for 20 s and then annealing at 80 °C for 30 min. Third, 90 nm-thick copper source and drain electrodes were deposited by E-beam evaporation with a nickel shadow mask, and the channel width and length were 1000 μm and 100 μm, respectively. Finally, we dropped the channel precursor solution between the source and drain and turned on the spin coating machine immediately after the gap between the source and drain was completely covered by the solution, so as to form a flat channel film between the source and drain. Subsequently, the device was annealed at 80 °C for 20 min. It is worth noting that the channel precursor solution was filtered by a 0.22 µm PTFE syringe filter before spin coating, and the rotation speed of the spin coating was 3000 rpm. It is important to mention that the structure and preparation method of CuI TFT were completely consistent with those of Zn-doped CuI TFT.

### 2.4. Device of Measuring Chitosan Capacitance

First, the chitosan precursor solution was deposited on the FTO glass substrates by spin coating at 3000 rpm for 20 s and then annealing at 80 °C for 30 min. Subsequently, the top FTO electrode was deposited by radio-frequency magnetron sputtering with a nickel shadow mask, and the top electrode width and length were 1000 μm and 100 μm, respectively. The face-to-face area of a parallel-plate capacitor was 1000 μm × 100 μm.

### 2.5. Characterization Equipment

The compositions of Zn-doped CuI films were determined by X-ray diffraction (D8 Discover) and X-ray photoelectron spectroscopy (ESCALAB250Xi). The structural characterization of the Zn-doped CuI film was obtained by scanning electron microscopy (SEM450). The capacitance-frequency curve of the chitosan gate dielectrics was investigated by an impedance analyzer (Agilent 4294A). Electrical characterizations of Zn-doped CuI TFTs on FTO glass substrates were performed by a semiconductor parameter analyzer (Keithley 4200 SCS).

## 3. Results and Discussion

**Figure 1:**
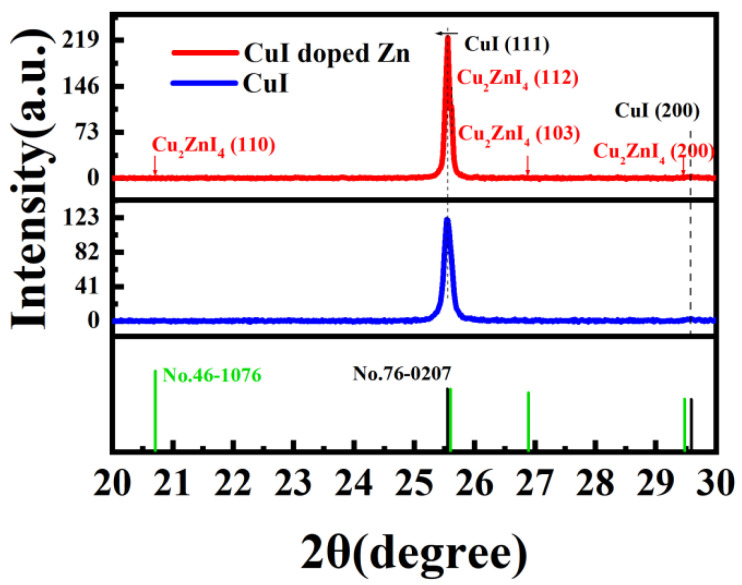
XRD patterns of CuI film and Zn-doped CuI film.

**Figure 2:**
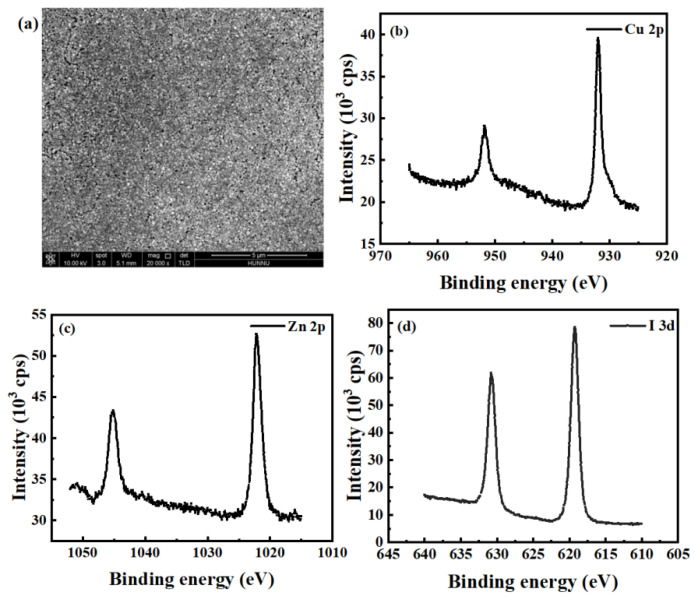
(**a**) SEM image of Zn-doped Cul film annealed at 80 °C; XPS spectra of (**b**) Cu 2*p*, (**c**) Zn 2*p* and (**d**) I 3*d* of the Zn-doped CuI film.

The X-ray diffraction (XRD) patterns of Zn-doped CuI thin films and pristine CuI thin films after 80 °C annealing are shown in Figure 1. Two Bragg reflection peaks were assigned to (111) and (200), corresponding to γ-phase CuI (JCPDS card No. 76-0207). The smaller Zn^2+^ (74 pm) with an ionic radius similar to Cu^1+^ (77 pm) can fully complement the Cu vacancy [23]. Since a small amount of Zn^2+^ was doped into CuI to fill or compensate for the Cu vacancies, the crystallinity of the material was improved, and the peak was elevated. Meanwhile, the original crystal structure was retained, and the smaller peak shift could be ignored due to the close ionic radii of Zn^2+^ and Cu^1+^. The SEM image of Zn-doped Cul film annealed at 80 °C is shown in Figure 2a. X-ray photoelectron spectroscopy (XPS) was performed to calculate the elemental composition of the samples on the Zn-doped CuI film surface. Figure 2b–d shows the core-level spectra of Cu *2p*, Zn *2p,* and I *3d*, respectively, confirming CuI:Zn was that only the substance present in the film.

**Figure 3:**
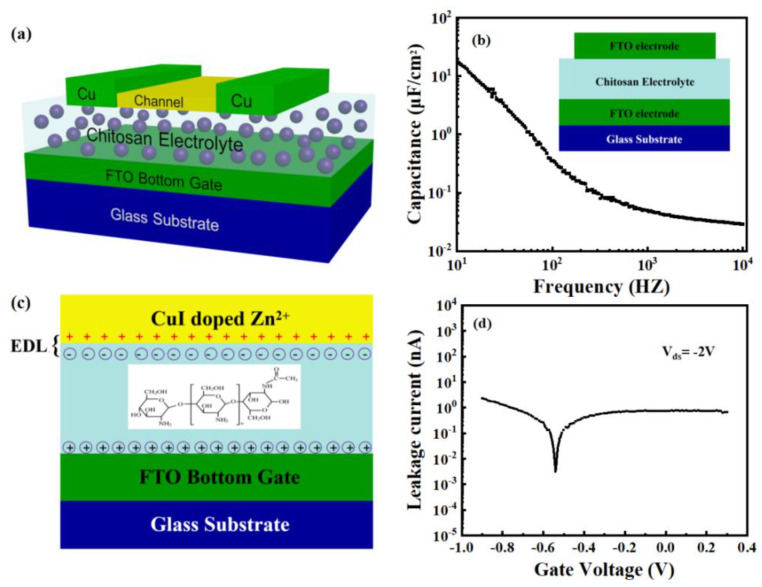
(**a**) Schematic diagram of the Zn-doped CuI TFTs on glass substrate. (**b**) Specific capacitance variation with frequency of the chitosan film (inset: FTO/chitosan/FTO device structure for capacitance measurement). (**c**) Schematic diagram of the EDL formation. (**d**) Gate leakage current of chitosan film.

**Figure 4:**
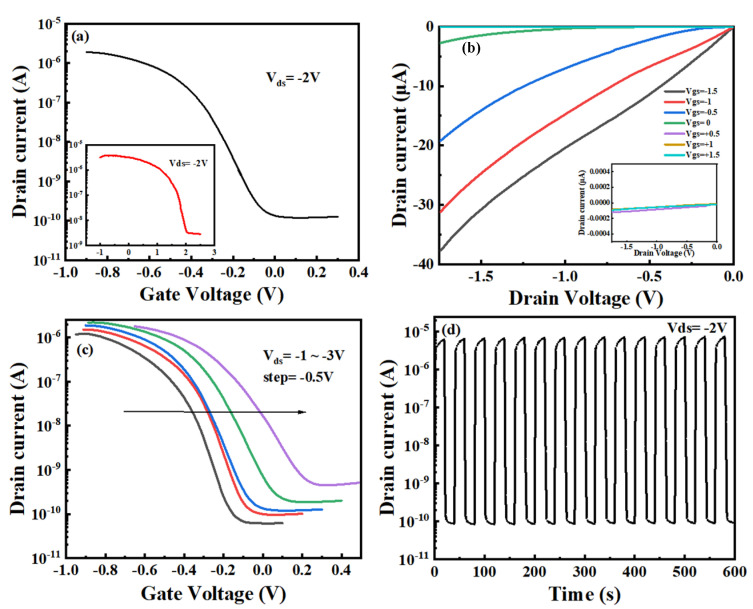
Electrical characteristics of Zn-doped CuI TFTs (**a**) Transfer characteristics (inset: transfer curves of CuI TFTs). (**b**) Output characteristics (inset: curves that cannot be fully displayed due to low drain current). (**c**) Transfer characteristics at different V_ds_. (**d**) Pulse response of the Zn-doped CuI TFTs to a square-shaped V_gs_ with a pulsed amplitude of V_+_ = 0.5 V and V_−_ = −1 V.

The schematic diagram of the Zn-doped CuI TFTs is shown in Figure 3a. Figure 3b shows the specific capacitance variation with frequency of the chitosan film at room temperature. It can be seen that the chitosan film exhibits a high specific capacitance, and the capacitance of the chitosan film decreases as the frequency increases. At 10 Hz, a maximum specific capacitance of 17.3 μF/cm^2^ is attained. Chitosan with water addition showed significantly higher ionic conductivity (10^−5^ S·cm^−1^) compared to dried chitosan (10^−9^ S·cm^−1^), which led to the formation of EDL that caused the device to work at low voltage [24,25]. The schematic diagram of the EDL formation is shown in Figure 3c. Due to the addition of deionized water to the chitosan precursor, a part of the free amino group in the chitosan gate dielectric film is deprotonated, and hydroxide ions are formed (-NH^2^ + H_2_O → NH^3+^ + OH^−^). During the preparation of chitosan precursor solution, the introduction of acetic acid provides a large number of protons, and these movable protons promote the protonation process. According to the Glotus mechanism, when a voltage is applied to the gate electrode, the movable protons in the chitosan film can be transferred from one oxygen atom to another through hydrogen bonds. The free hydroxide ion increases the conductivity of the chitosan gate dielectric film, which leads to a larger capacitance. When an appropriate negative voltage is applied to the gate electrode, a large number of protons are gathered near the gate, and the negative ions in the chitosan membrane are repelled to the side near the channel. Equal-density hole carriers are induced in the channel to realize the formation of EDL. The gate leakage current of the chitosan film is shown in Figure 3d at a bias voltage of −2 V. A low gate leakage current of less than 2.5 nA was produced, which is three magnitude orders less than the channel current and substantially less than that of solid polymer electrolytes or ionic liquids, demonstrating that the influence of leakage current during device operation could be ignored [26,27]. Figure 4a shows the typical transfer characteristics of Zn-doped CuI TFTs. The response time of the transistor was 1.3875 s during the measurement. The on/off current ratio I_on/off_, subthreshold swing SS, and threshold voltage V_th_ are estimated to be 1.58 × 10^4^, 70 mV/decade, and −0.2 V, respectively. However, the I_on/off_ value of the pure CuI TFT was below 10^3^ under the same conditions, as shown in the inset of Figure 4a. The on-current of CuI TFT is slightly larger than that of Zn-doped CuI TFT, which is caused by scattering of Zn ion [28,29]. In addition, the solution-processed method is difficult to realize the patterned channel. In the process of deposition channel, there is part of residual channel solution near the source electrode and the drain electrode. An edge electric field can create a parasitic path in the source-drain conduction near the electrode edges [30,31]. The hole mobility decreases as Zn ions replaced or supplemented Cu vacancies, which weakens the channel conductivity [17,32]. The decrease in electrical conductivity of channel solution contributes to the decrease in edge electric field. The smaller edge electric field weakens the unexpected contribution of the parasitic path and reduces the off-current of the TFT obviously [31]. On the whole, the I_on_/I_off_ of the device doped with Zn ions is larger than CuI TFT. The threshold voltage of CuI TFT is 2.02 V. The subthreshold swing SS of CuI TFT is 1.05 V/decade. The field-effect mobility (μ = [2LI_ds_]/[(WC_i_)(V_gs_−V_th_)^2^]) of Zn-doped CuI TFT is estimated to be 0.40 cm^2^V^−1^s^−1^, where L is the channel length, W is the channel width, and C_i_ is the specific gate capacitance at 10 Hz (C_i_ = 17.3 μF/cm^2^). Figure 4b shows the corresponding output characteristics of the Zn-doped CuI TFT. The V_gs_ ranged from −1.5 V to 1.5 V in 0.5 V steps. As shown in Figure 4c, as the transfer curve shifts to the right with V_ds_ from −1 V to −3 V in −0.5 V steps, the values of V_th_ are −0.309 V, −0.236 V, −0.205 V, −0.112 V, and −0.051 V, respectively. It was shown that effective electrostatic coupling is performed between the source and drain electrodes and the bottom gate [24]. Figure 4d shows the transient response of the Zn-doped CuI TFTs driven by period square wave pulses of V_gs_ = 0.5 V to −1 V at V_ds_ = −2 V. The device shows prominent reproducibility of the current response in the control of regular pulsed V_gs_. The good electrical properties of the Zn-doped CuI TFT provide the necessary foundation for the application of the device.

**Figure 5:**
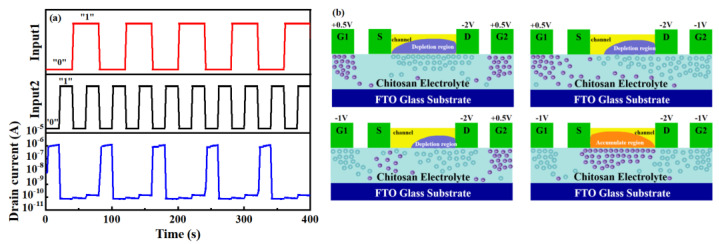
(**a**) NOR logic of such p-channel TFTs. (**b**) Schematic diagram of the p-channel TFTs with NOR logic.

In addition, symmetric Zn-doped CuI TFTs were fabricated with two in-plane gates. As demonstrated in Figure 5a, the NOR logic operation was performed on one single p-channel TFT. G1 and G2 are defined as terminals of input1 and input 2, respectively. The positive input signal (+0.5 V) was defined as logic 1, and the negative input signal (−1 V) was defined as logic 0. The channel current was defined as the output. The channel current was blocked if either or both inputs were logic 1. The channel current was high (logic 1) when the input signals are both negative (logic 0). The resilient operation of this logic gate was demonstrated through the on/off current ratio, which was maintained at 10^4^ between the two logic states. The schematic diagram of the NOR logic is shown in Figure 5b. When +0.5 V is applied to both G1 and G2, protons are attracted close to the channel and form the Helmholtz layer, and image charges with an opposite sign and equal density are induced in the channel on the side close to the chitosan electrolyte. The channel was completely depleted, and the device was turned off. With a +0.5 V and −1 V bias applied to G1 and G2, respectively, the depleted region in the channel narrowed and the device remained closed. When −1 V is applied to both G1 and G2, most of the protons in the chitosan electrolyte are gathered around the drain electrode and the gate electrodes which induce many holes to form accumulation areas in the channel. The TFT was in an on state in these circumstances.

**Figure 6:**
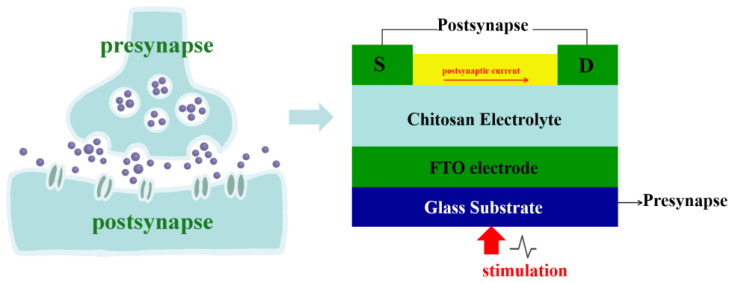
Schematic diagram of Zn-doped CuI TFTs simulating synaptic neurons.

**Figure 7:**
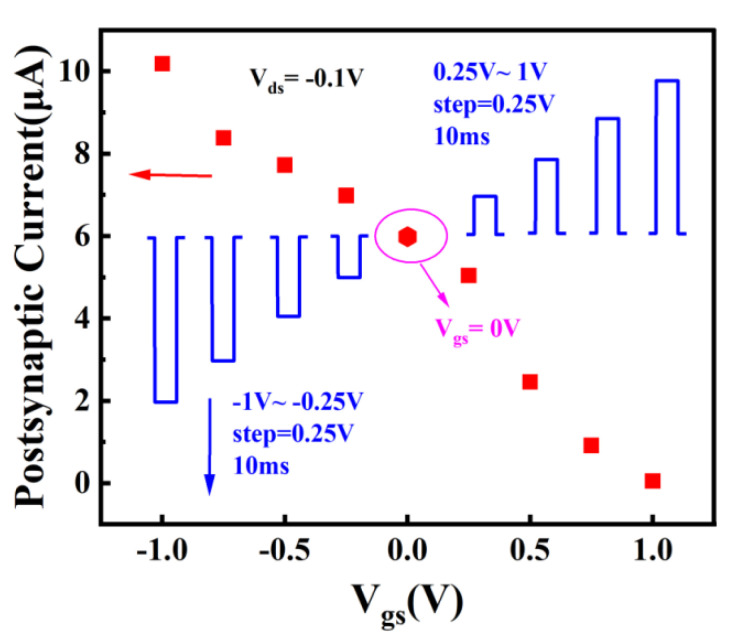
Postsynaptic current responses motivated by different stimuli based on Zn-doped CuI TFTs.

Figure 6 shows the schematic diagram of Zn-doped CuI TFTs simulating synaptic neurons. Presynaptic cells transmit information to postsynaptic cells through chemical signals or electrical signals. Synaptic types include excitatory synapses and inhibitory synapses. The direction of information transmission between neurons is unidirectional. When neurons are stimulated, the permeability of the presynaptic membrane to calcium increases, which induces some synaptic vesicles of presynaptic neurons to closely fuse with the presynaptic membrane. The synaptic vesicles ruptured near the presynaptic membrane, and neurotransmitters were released into the synaptic space. Neurotransmitters spread to the postsynaptic membrane and bind to protein receptors, changing the ion permeability of the postsynaptic membrane. Excitation or inhibition of the postsynaptic membrane produces an EPSC or an IPSC, respectively. In our devices, the gate voltage was considered to be the stimulation of presynaptic neurons. Channel currents were defined as postsynaptic currents. The postsynaptic current was regulated by the presynaptic electrode (gate electrode). In the whole process, the signal processing and transmission were parallel, which is particularly similar to the processing of neural morphological information. As shown in Figure 7, the blue pattern represents a model of different pulse. The purple circle emphasizes the postsynaptic current when the pulse intensity is 0 V. When the presynaptic electrode is stimulated by −1 V to −0.25 V in a step of 0.25 V for 10 ms, the postsynaptic membrane is excited and a significant EPSC is produced (the resting potential of the presynaptic electrode is 0 V). EPSC increases with the absolute value of the difference between presynaptic electrode voltage and resting potential. In addition, when the presynaptic electrode is stimulated by 0.25 V to 1 V in a step of 0.25 V for 10 ms, the postsynaptic membrane produces an obvious IPSC (the resting potential of the presynaptic electrode was 0 V). IPSC also increases with the absolute value of the difference between presynaptic voltage and resting potential. More importantly, the whole curve of gradual synaptic response is almost linear.

**Figure 8:**
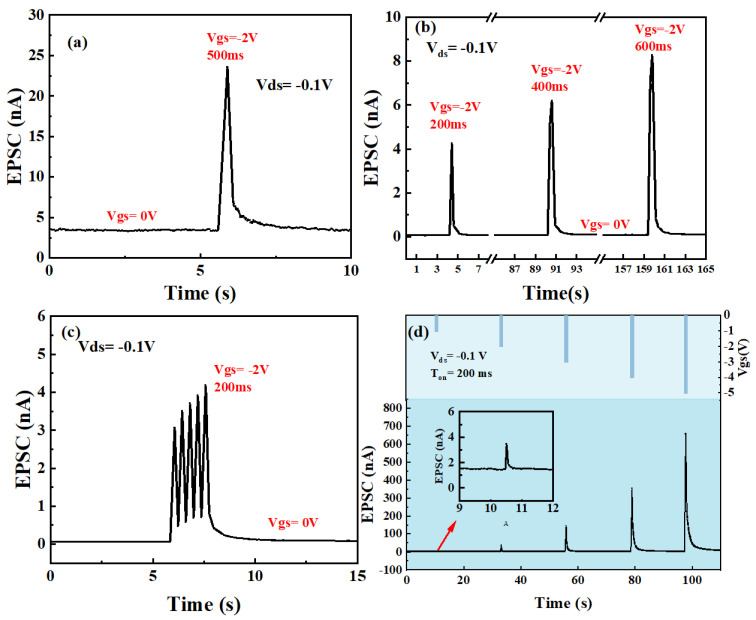
Schematic diagram of pain perception of the human brain based on Zn-doped CuI TFTs (**a**) EPSC of a single stimulus. (**b**) EPSC with different stimulation times (**c**) EPSC under five consecutive stimuli. (**d**) EPSC with different stimulus intensities.

Subsequently, simple human brain pain nerve conduction was simulated on this p-type Zn-doped CuI TFT. The schematic diagram of pain perception in the human brain is shown in Figure 8. The V_gs_ is defined as the intensity of stimulation to the human brain. Channel currents are defined as EPSC produced after stimulation. As shown in Figure 8a, a stimulus was applied to the human brain (500 ms, −2 V), and then ~24 nA EPSC was produced immediately. After stimulation, EPSC decreased rapidly at first and then slowed down until the memory of pain almost disappeared. As shown in Figure 8b, the ~4 nA EPSC was generated when the first stimulus is applied (200 ms, −2 V). After the pain memory almost disappeared, ~6 nA EPSC was produced by applying a second stimulus (400 ms, −2 V). After the second pain memory almost disappeared, a third stimulus (600 ms, −2 V) was applied to produce a larger ~8 nA EPSC. Obviously, the EPSC produced by a stimulus is closely related to the stimulation time. With the increase in stimulation time, the larger the postsynaptic current produced, the stronger the corresponding pain. As shown in Figure 8c, a larger EPSC was formed by multiple consecutive stimuli at short intervals. The EPSC produced by the latter was slightly higher than that of the former when five stimuli are applied successively in a short period of time, which was caused by the relaxation effect of mobile ions in chitosan. After a stimulus was applied to the presynaptic electrode (gate) (200 ms, −2 V), the ions in chitosan moved rapidly due to the electric field. A large number of carriers are induced in the channel because of the EDL effect. After the stimulation, the movable ions of the chitosan film will slowly return to their initial state. However, if another stimulus is applied before the ion has fully returned to the initial state, more carriers will be induced in the channel and a larger channel current will be obtained. It reflects that the pain could be more severe when the brain is stimulated again before the pain memory disappears. In addition, the relationship between stimulus intensity and human brain pain perception is shown in Figure 8d. The experiment confirmed that a higher intensity of stimulation leads to a higher EPSC value. The larger the intensity of stimulation to the brain, the longer the pain memory is.

## 4. Conclusions

In summary, Zn-doped CuI TFTs gated by chitosan film were fabricated at a low temperature. The XRD characterizations of CuI film and Zn-doped CuI film were compared, which evidenced that the Zn ions did not change the crystal structure of CuI. However, the substitution or supplementation of copper vacancies by Zn ions elevates the electrical performance of TFTs. A high specific capacitance of the chitosan film was calculated to be 17.3 μF/cm^2^, which prompted the TFTs to work at low voltage. The threshold voltage of Zn-doped CuI TFT is −0.2 V. Such TFTs showed significant electrical behavior. The on/off current ratio, field effect mobility, and subthreshold swing were estimated to be 1.58 × 10^4^, 0.40 cm^2^V^−1^s^−1^, and 70 mV/decade, respectively. Moreover, NOR logic was successfully demonstrated on the basis of one single Zn-doped CuI TFT. More importantly, synaptic neuron simulation was realized on this p-channel TFT for the first time. Based on the Zn-doped CuI TFTs, EPSC and IPSC are realized, and the whole postsynaptic current is linear. In addition, a simple pain perception system of the human brain is demonstrated. Zn-doped CuI TFTs are favorable for the evolution of next-generation low-power electronics, complementary electronic circuits, and biosensors.

## Data Availability

The data that support the findings of this study are available on request from the corresponding author.
